# Homoharringtonine and omacetaxine for myeloid hematological malignancies

**DOI:** 10.1186/1756-8722-7-2

**Published:** 2014-01-03

**Authors:** Shuqing Lü, Jianmin Wang

**Affiliations:** 1 Department of Hematology, Changhai Hospital, Second Military Medical University, 168 Changhai Road, Shanghai 200433, China

**Keywords:** Homoharringtonine, Omacetaxine, Chronic myeloid leukemia, Acute myeloid leukemia, Myelodysplastic syndrome

## Abstract

Homoharringtonine (HHT), a plant alkaloid with antitumor properties originally identified nearly 40 years ago, has a unique mechanism of action by preventing the initial elongation step of protein synthesis. HHT has been used widely in China for the treatment of chronic myeloid leukemia (CML), acute myeloid leukemia (AML) and myelodysplastic syndrome (MDS). Omacetaxine, a semisynthetic form of HHT, with excellent bioavailability by the subcutaneous route, has recently been approved by FDA of the United States for the treatment of CML refractory to tyrosine kinase inhibitors. This review summarized preclinical and clinical development of HHT and omacetaxine for myeloid hematological malignancies.

## Background

The genus Cephalotaxus comprises nine species, which are mostly concentrated in China, but are also found in eastern India, Thailand, the Korean peninsula and Japan. The anti-inflammatory and antiparasitic effects of Cephalotaxus fortunei Hook plants have been used in Chinese folk remedies for a long time and its antineoplastic effects have also been studied [1]. Paudler et al. [2] isolated harringtonine and cephalotaxine from Cephalotaxus harringtonia in 1963 for the first time. In 1969, Powell et al. [3,4] determined their structures and confirmed the antileukemic effects on mouse P-388 and L-1210 lines of some ester alkaloids isolated from Cephalotaxus harringtonia: harringtonine, isoharringtonine, deoxyharringtonine, and homoharringtonine (HHT). HHT differs from harringtonine in that it has a methylene group inserted in the side chain. Chinese scientists conducted research that confirmed the anti-leukemia effects of harringtonine and HHT in patients with acute myeloid leukemia (AML) and chronic myeloid leukemia (CML). In most of those studies, a racemic mixture of harringtonine and HHT was used. Despite similar chemical and preclinical activities, HHT was chosen over harringtonine because of its better extraction yield from its source, *Cephalotaxus harringtonia*[5-7]. A series of studies conducted in the United States confirmed the utility of this agent for CML [8,9]. Since then, harringtonine and HHT have been widely used in the treatment of CML, AML and myelodysplastic syndrome (MDS), especially in China [10-13]. However, the clinical development of HHT in CML stopped with the discovery and popularization of the tyrosine kinase inhibitor (TKI), imatinib mesylate (Gleevec) [14]. Recently, the interest in HHT for CML has been encouraged by positive results in patients who failed on imatinib therapy.

The natural purification of harringtonine and HHT has caused significant damage to the environment. In 1999, Robin et al. [15] reported, for the first time, the synthesis of semisynthetic HHT (sHHT). sHHT involves the direct esterification of cephalotaxine extracted from dry leaves of cephalotaxus, not from the bark. Only one 70th of the amount of cephalotaxus is required to extract sHHT compared with its natural counterpart, and it is also purer (99.7%). In addition, sHHT has excellent bioavailability by the subcutaneous (SC) route. sHHT is known currently as omacetaxine mepesuccinate (ceflatonin, CGX-653, Myelostat) and is being developed by ChemGenex Pharmaceuticals Ltd. (Menlo Park, CA, USA), in collaboration with Stragen Pharma (Geneva, Switzerland). Omacetaxine has recently been proved by FDA of the United States as an orphan drug to treat CML patients resistant to TKIs. In this paper, we will review the unique mechanism of action, and the development of HHT and omacetaxine for the treatment of hematological malignancies.

### Mechanisms of action and preclinical studies

Harringtonine and HHT inhibit protein translation by preventing the initial elongation step of protein synthesis via an interaction with the ribosomal A-site [16,17]. Recent crystallographic studies have shown that HHT blocks protein synthesis by competing with the amino acid side chains of incoming aminoacyl-tRNAs for binding to the A-site cleft in the peptidyl transferase center of the ribosome [18]. HHT leads to a general decrease in synthesis efficiency of all proteins. An important short-term effect of HHT on cells is the rapid loss of proteins with short half-lives. A number of proteins related to cell survival and proliferation with short half-lives are encoded by mRNAs that possess complex 5′ UTRs that are G/C rich and have complex 3-dimensional structures (e.g. c-Myc, Mcl-1 and Cyclin D1). HHT and omacetaxine induce the rapid loss of a number of short-lived proteins from various cell lines of hematological malignancies. These short-lived proteins clearly regulate proliferation and cell survival and their loss is likely to be involved in the apoptosis induced by HHT and omacetaxine. An early event that triggers HHT- and omacetaxine-induced apoptosis is the downregulation of Mcl-1, which was originally identified as an antiapoptotic Bcl-2 family protein during differentiation of myeloid cells. These effects were replicated in primary cells obtained from patients with AML and patients with CML. Mcl-1 downregulation may result in an increase in free BH3-only proteins, such as Bim, tBid, Bik, and Puma, in addition to reducing the levels of beta-catenin and X-linked inhibitor of apoptosis (XIAP) proteins [19-24]. The short-lived protein c-Myc can promote expression of elongation initiation factor 4 F (eIF-4 F) proteins, which feed forward to promote translation of mRNAs that possess complex 5′ UTRs including c-Myc. As c-Myc is preferentially lost from cells treated with HHT, levels of mRNAs encoding eIF-4 F proteins are likely to be rapidly reduced and augment the effects of downregulation of protein translation initiation [25,26].

In vitro studies showed that HHT could induce apoptosis of AML and MDS cells via upregulation of pro-apoptotic bax and downregulation of the protein inhibitor survivin [24,27,28]. Moreover, a study by Tong et al. showed that HHT might act as a broad-spectrum protein tyrosine kinase inhibitor that inhibits the phosphorylation of the signal proteins by oncogenic proteins such as JAK2V617F, Bcr-Abl, thus blocking the survival and proliferative signal pathway of primary AML cells and AML cell lines such as HEL, K562 and HL-60 cells [29].

**Figure 1:**
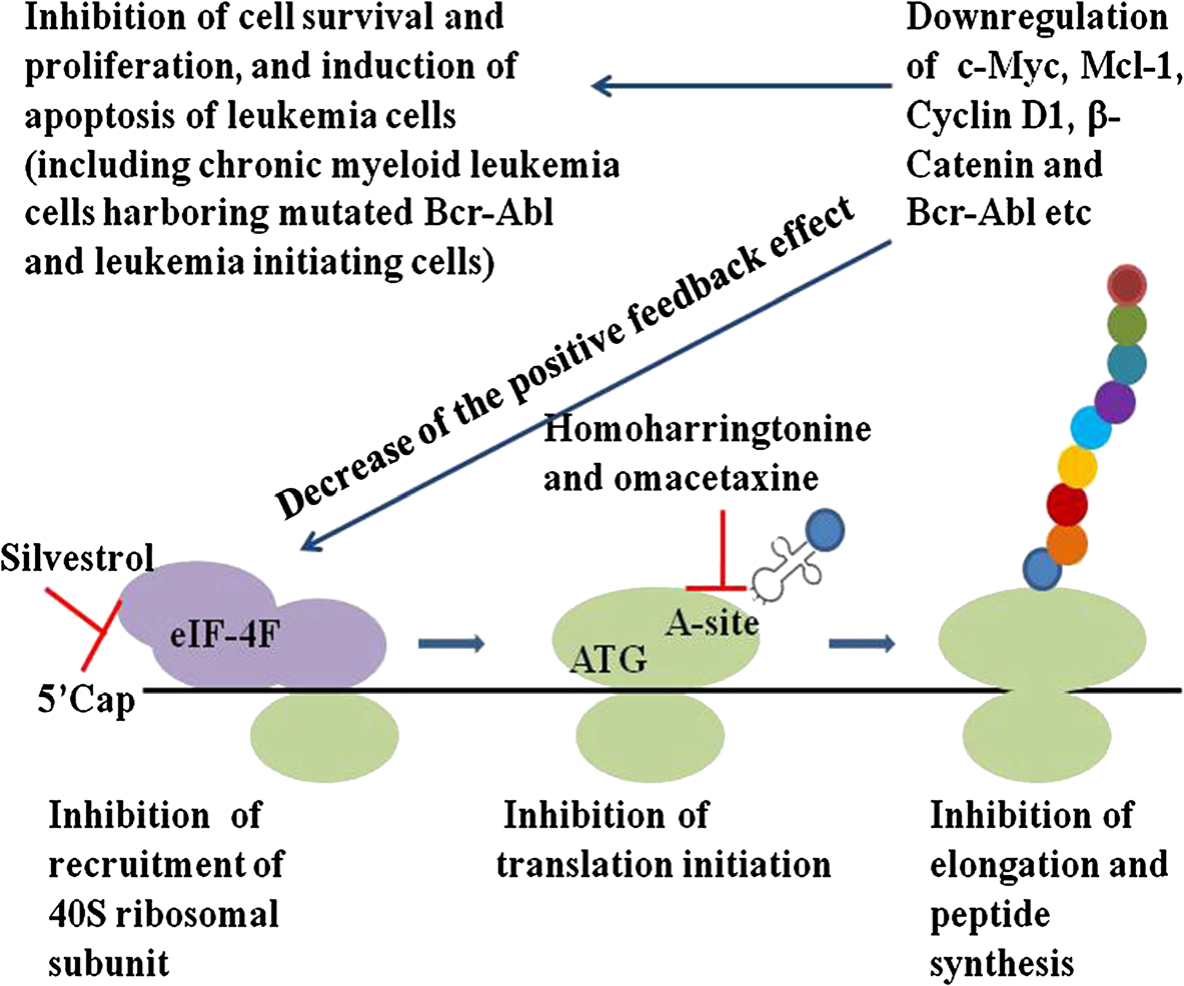
The mechanisms of the protein translation inhibitors homoharringtonine and silvestrol.

This effect of HHT is similar to other novel protein translation inhibitors, such as silvestrol. However, the mechanisms of these protein translation inhibitors are different (Figure 1). Silvestrol is a cyclopenta benzofuran rocaglate isolated from the Indonesian plant *Aglaia foveolata*, which has a unique dioxanyl ring-containing side chain. Silvestrol interferes with the assembly of the eIF4F translation complex by promoting an aberrant interaction between capped mRNA and eIF4A, thereby blocking protein synthesis at the initiation step. This inhibition of protein synthesis by silvestrol also results in a preferential depletion of proteins with short half-lives, such as Mcl-1, Cyclin D1 and c-Myc. Silvestrol was reported to have activity against leukemia cells in vitro and in vivo [30,31].

Interestingly, HHT has proved synergistic with other agents active in CML, such as IFN-a, cytosine arabinoside (Ara-C), or both combined. The combination of all three agents was highly active against leukemic cells from patients with CML in the chronic phase (CML-CP) [32]. Protein translation of mRNAs with complex 5′ UTRs in Bcr-Abl positive cells is upregulated via Bcr-Abl-mediated activation of phosphoinositide-3-kinase (PI3K)/AKT/mammalian target of rapamycin (mTOR) signaling pathways [33]. The inhibition of Bcr-Abl by imatinib markedly reduced protein translation initiation. Imatinib interacts synergistically in inducing apoptosis of Bcr-Abl positive cells with compounds that interfere with translation directly or regulate protein translation initiation, which includes HHT and omacetaxine. HHT and omacetaxine also reduce Bcr-Abl protein levels in Bcr-Abl positive cells [34,35]. Synergy was also observed when HHT and imatinib were used in combination against imatinib-resistant cell lines and against primary blastic cells obtained from patients with advanced phase CML (CML-AP) [36]. HHT does not compete with ATP at the catalytic domain of the Bcr-abl kinase; therefore, it is conceivable that the activity of HHT against Bcr-Abl positive cells is independent of their Bcr-Abl mutational status. In fact, in vitro data have demonstrated that the activity of HHT against Bcr-Abl positive cells was similar irrespective of whether the cells harbored non-mutated Bcr-Abl or the imatinib-resistant E255K or T315I mutations [19]. These studies raise the possibility that the efficacy of current CML therapy with TKIs may be increased by combined treatment with HHT and omacetaxine.

Leukemia initiating cells (LICs) are a population of stem cells that are capable of tumor initiation and maintenance of the disease. LICs in CML are thought to reside in a population of Bcr-Abl positive cells with characteristics of hematopoietic stem cells. Current TKIs do not kill these cells at a high frequency, but rather cause apoptosis in more differentiated Bcr-Abl positive cells of myeloid and lymphoid lineages [37]. In recent years, many studies have shown that HHT and omacetaxine could effectively kill Bcr-Abl positive LICs in vitro and in a mouse model of CML. The reason why HHT and omacetaxine target Bcr-Abl positive LICs may be that Bcr-Abl positive LICs require expression of certain short-lived proteins (e.g. Mcl-1, β-Catenin and the β subunit of IL-3R). These proteins are preferentially lost to induce apoptosis and impair the renewal of Bcr-Abl positive LICs after treatment with HHT or omacetaxine [35,38-41]. A recent study by Shen et al. showed HHT could effectively kill the LICs in the human AML cell line KG1 by inhibiting cell growth and inducing apoptosis, which was associated with activation of the caspase pathway and downregulation of anti-apoptotic protein Bcl-2 and phosphorylated-Akt [42].

### HHT clinical development in CML

The initial clinical trials of cephalotaxine esters in patients with cancer were conducted in the 1970s [1,5-7]. Nine of 15 patients with CML treated with HHT (5 mg d^-1^ ~ 7 mg d^-1^ for 7 ~ 10 days) achieved complete hematological remission (CHR) [7]. In a subsequent study, 39% (32 of 82) of CML patients treated with HHT (intravenously, 4 mg d^-1^ to 8 mg d^-1^) achieved CHR [10]. Huang et al. reported that 57.6% of 33 CML-CP patients treated with harringtonine (intravenously, 4 mg d^-1^, dose reduction and length of the course according the WBC counts) during 1991 ~ 1995 achieved CR [43]. In a study carried out during 1996 ~ 2002, 76 newly-diagnosed CML-CP patients were treated with HHT (intravenously, 1.5 mg m^-2^ daily for 7 ~ 11 d every month). Among 55 patients with cytogenetic data, 38.2% achieved CyR (cytogenetic response) and 20% achieved MCR (major cytogenetic response), while only 2 of 10 patients with cytogenetic data achieved minor cytogenetic response in the group treated with hydroxyurea. The estimated 4-year overall survival (OS) was 46.2%, which was significantly higher than that of the group treated with hydroxyurea (27%, 10/27) [44]. In 2008, Li et al. reported a low-dose and long term protocol of HHT (intravenously or intramuscularly, 1 mg d^-1^ for 8 weeks or 2 mg d^-1^ for 4 weeks, next cycle beginning after 4–5 weeks interval until 4 years), which resulted in a CHR of 66% (27 of 41) and with 5-year progression-free survival rate (PFS) 95% (39 of 41) [45].

The first two phase I studies performed in the United States was published in 1983 and 1984, in which, a highly purified form of HHT was administered daily for 1 to 10 days, with dose escalation from 0.2 mg m^-2^ to 8 mg m^-2^ daily. Cardiovascular collapse (hypotension and tachycardia) happened in approximately 25% of patients who received HHT at doses of 5 mg m^-2^ or 6 mg m^-2^ daily, which were occasionally fatal. The short infusion maximum tolerated dose (MTD) was <3 mg m^-2^ to 4 mg m^-2^ intravenously over 1 hour daily for 5 consecutive days [8,46]. About 10 years later, a study performed by O’Brien et al. showed encouraging results. HHT was given as a single agent to 71 patients with late CML-CP at a dose of 2.5 mg m^-2^ daily for 14 days during the remission induction phase and for 7 days monthly during the maintenance phase. Seventy-two percent of 58 assessable patients achieved CHR and 31% of 71 patients achieved a CyR, including 15% MCyR and 7% complete cytogenetic response (CCyR). The major toxicities were myelosuppression which occurred in 39% of induction courses [11].

Subsequently, HHT was administered to 99 patients with early CML-CP using a dose schedule similar to that of the previous study of O’Brien et al., for six cycles, followed by the administration of IFN-a maintenance. The results showed that the rates of CHR, CyR and MCyR were 92%, 60% and 27%, respectively, which were superior to those in historic control patients after 6 months of IFN-a therapy [11]. In another study performed by Kantarjian et al., the combination of HHT and low-dose ara-C was used to treat 100 patients in late CML-CP who had failed on IFN-a therapy. Seventy-two percent of patients achieved CHR, and 32% achieved CyR, including 15% MCyR and 5% CCyR [47]. In a phase II study reported by Stone et al., the combination of HHT (2.5 mg m^-2^ daily) and ara-C (7.5 mg m^-2^ daily), given by continuous intravenous infusion for 7 days every 28 days, was administrated to 44 patients with newly-diagnosed CML-CP. The results showed an 82% CHR and a 17% MCyR [48]. Moreover, O’Brien et al. treated 90 patients in early CML-CP with the triple combination of HHT, IFN-a, and low-dose ara-C, which yielded a 94% CHR and a 74% CyR, including 22% CCyR. After a median follow-up of 46 months, the estimated 5-year OS rate was 88%, and only 9% patients had progressed to CML-BP [49]. In China, He et al. treated seven CML-CP patients with the combination HHT and AS2O3 (10 mg d^-1^ for 2 ~ 3 weeks, HHT 3 ~ 4 mg d^-1^ for 1 ~ 2 weeks). After the first course treatment, four patients achieved CHR [50]. These studies suggest that HHT-based combination therapy results in improved clinical outcomes compared with single-agent HHT in patients with CML-CP.

The striking results obtained by TKIs impaired the development of HHT in CML. However, the distinct mechanisms of action and the remarkable effects of HHT on Bcr-Abl positive LICs and imatinib-resistant Bcr-Abl mutants (including T315I) in vitro, led to the return of HHT to CML therapy. Notably, the T315I Bcr-Abl mutation does not respond to any approved TKI in vitro or clinically, except ponatinib which was approved by US FDA more recently [51]. The prognosis for chronic-phase CML patients with this mutation is poor. In a Phase I/II study, patients with CML who had achieved CyR but achieved a plateau in Bcr-Abl transcripts after treatment with imatinib for at least 2 years were given omacetaxine (1.25 mg m^-2^ twice daily for 1–3 days every 28 days). Of 10 evaluable patients, seven patients, including two with the Bcr-Abl mutation, had an appreciable decline in Bcr-Abl transcript levels. The results suggested the addition of omacetaxine should be considered for patients on imatinib who fail to obtain low levels of minimal residual disease [52]. In another Phase I/II study, six imatinib-resistant CML patients, including two patients with Bcr-Abl mutations, were treated with omacetaxine alone (2.5 mg m^-2^ intravenously over 24 hours, followed by 1.25 mg m^-2^, subcutaneously, twice a day, for 14 days in inducing period and for 7 days in maintenance period every month). CHR was obtained in all five evaluable patients and three had CyR, including one with CCyR. The Bcr-Abl mutations in both instances became undetectable [53]. In 2007, Legros et al. reported that Bcr-Abl (T315I) transcript disappeared in an imatinib-resistant CML patient treated with omacetaxine for the first time [54]. A study performed by Nicolini et al. investigated the effects of omacetaxine on non-mutated and T315I-mutated Bcr-Abl transcripts in eight TKI-resistant CML-CP patients. An initial rapid decline and a sustained disappearance of T315I-mutated transcripts were observed in 50% of the patients. As the non-mutated leukemic burden reduction was modest, two patients were submitted to nilotinib after 9 months of sustained Bcr-Abl T315I transcripts negativity on omacetaxine: the mutated transcripts remained undetectable after a median follow-up of 12 months on nilotinib challenge [55]. In a recently reported phase II study, the efficacy of omacetaxine in CML-CP patients with T315I after TKI failure was assessed. Patients received omacetaxine 1.25 mg m^-2^ twice daily for 14 days every 28 days in induction period and for 7 days every 28 days in maintenance period. Seventy-seven percent of 62 patients achieved CHR, 23% achieved MCyR, including 16% with CCyR [56]. These results suggested that HHT and omacetaxine might provide an effective treatment for CML patients with the T315I mutation. HHT and omacetaxine (a non-targeted therapy) might provide better disease control, allowing the disappearance of the mutated clone, probably elicited by the clone deselection after TKI release, and could allow for a safe TKI rechallenge in patients with resistant CML-CP. In consideration of the effect of HHT and omacetaxine on the LICs, the combination treatment of HHT or omacetaxine with IM in newly diagnosed CML may provide an approach to cure the disease and reduce the risk of relapse after the termination of IM treatment.

### HHT clinical development in AML

After the initial clinical trials of harringtonine and HHT in leukemia patients [1,5-7], harringtonine and HHT was widely used in China to treat patients with AML. For example, in 1989, Wang et al. reported the 40% (8/20) of patients with AML (16 newly-diagnosed, 4 relapsed, 20 ~ 76 years old) achieved CR after induction of low-dose harringtonine (0.6 ~ 1 mg d^-1^ for 20 days or until CR or until severe adverse reaction) [57]. At the same time, Huang et al. reported a 75% (12/16) CR rate of AML patients treated with low-dose HA regimen (HHT 1 ~ 2 mg d^-1^, Ara-c 7.5 ~ 15 mg 12 h^-1^ until CR or severe myelosuppression) [58]. These promising results encouraged a series of studies of a 7-day HA induction regimen for the treatment of AML. For example, Zheng et al. treated 34 AML patients (31 newly diagnosed and 3 relapsed) with HA regimen (harringtonine 3 mg d^-1^, for 3 days, Ara-c 150 ~ 200 mg d^-1^ for 7 days, intravenously) from 1986 to 1988, the CR rate was 70.6% [12]. In a random control study for newly-diagnosed AML patients performed by Bian et al., the CR rate of the HA regimen (HHT 3 ~ 6 mg d^-1^ for 7 days, Ara-c 100 ~ 300 mg d^-1^ for 7 days) was 60.7% (51/84) and that of DA (daunorubicin (DNR) 40 ~ 80 mg d^-1^ for 3 days, Ara-c 100 ~ 300 mg d^-1^ for 7 days) regimen was 68.9% (40/58). After several regimens alternate consolidate therapy, the 5-year OS rates of the HA group and the DA group were 32% and 28%, respectively. The differences of near-term CR rates and long-time OS rates were not significant between the HA regimen and the DA regimen [59]. In the study performed by Fu et al., the CR rates of newly-diagnosed AML patients treated with the HA regimen (HHT 2 ~ 4 mg d^-1^ for 5–7 days, Ara-c 100 ~ 200 mg d^-1^ for 7 days) and the DA regimen (DNR 40 ~ 60 mg d^-1^ for 3 days, Ara-c 100 ~ 200 mg d^-1^ for 7 days) were comparable (73.14%, 30/41 *vs.* 78.46%, 51/65) [60]. In the study of Yang et al., 56 newly diagnosed AML patients randomly received HA or DA treatment. The CR rates in the HA group (63.6%) and the DA group (67.6%) were also similar. The adverse reactions to HA were relatively mild [61]. Though lacking data of multiple-centre, random, controlled study, these studies could suggest that HA is an effective induction regimen, comparable with DA regimen, for AML patients (Table 1). HHT was also well tolerated and did not cause more serious adverse events than DNR in the induction of AML (Table 2).

**Table 1:** The summary of studies of homoharringtonine-based regimens in the induction of acute myeloid leukemia

**Reference**	**Regimen**	**Patients no.**	**CR, %**	**3-year OS, %**
Huang 1989 [58]	Low dose HHT + low Ara-c	16	75.0	NP
Zheng 1989 [12]	HHT + Ara-c	34	70.6	NP
Bian 1993 [59]	HHT + Ara-c	84	60.7	32 (5-year OS)
Fu 2001 [60]	HHT + Ara-c	41	73.1	NP
Yang 2005 [61]	HHT + Ara-c	56	63.6	NP
Xue 1995 [62]	HHT + Ara-c + DNR	50	86.0	NP
Xiao 2008 [63]	HHT + Ara-c + DNR	72	86.1	55.9
Jin 2013 [67]	HHT + Ara-c + DNR	198	67.0	48.0
Wan 1997 [64]	HHT + Ara-c + aclarubicin	25	76.0	NP
Song 2011 [66]	HHT + Ara-c + aclarubicin	150	81.0	45.0
Jin 2013 [67]	HHT + Ara-c + aclarubicin	206	73.0	48.5

*HHT* homoharringtonine, *Ara-c* arabinoside, *CR* complete remission, *OS* overall survival, *NP* not provided.

**Table 2:** Comparison of the toxicity of homoharringtonine and daunorubicin in the induction of acute myeloid leukemia

**Reference**	**Rigemen (NO.)**	**Infection (%)**	**Damage of liver function (%)**	**Cardiotoxicity (%)**
Yang 2005 [61]	DA(34)	44.4	13.9	8.8
	HA(22)	36.4	8.8	0
Yuan 2011 [72]	DNR + ATRA(61)	67.2	14.8	0
	HTT + ATRA(54)	57.4	18.5	1.9

*DA* daunorubicin + arabinoside, *HA* homoharringtonine + arabinoside, *DNR* daunorubicin, *HTT* homoharringtonine.

Subsequent studies also showed that an HHT based triple drug combination was highly effective in the treatment of AML. Xue et al. treated adult AML patients (newly diagnosed 38, relapse or refractory 12) with an HAD combination regimen (HHT 4 mg d^-1^, for 7 days, DNR 60 mg d^-1^ for 3 days, Ara-c 200 mg d^-1^ for 7 days). The result showed that the CR rate was as high as 86.0% (43/50), while the treatment related mortality (TRM) was only 4% [62]. Xiao and colleagues showed that in 72 young untreated patients, this HAD regimen resulted in a CR rate of 86.1%, and a 3-year OS rate of 55.9% [63]. In 1997, Wan reported an HAA regimen (HHT 3 mg d^-1^, for 3 days; Ara-c 200 mg d^-1^, for 7 days; aclarubicin 20 mg d^-1^, for 3 days) in the treatment of AML patients (20 newly diagnosed, five refractory or relapsed) and the CR rate was 76.0% [64]. The efficacy of the HAA regimen in the treatment of young (14–60 years old) de novo AML patients was confirmed in studies performed by Jin and colleagues [65,66]. The encouraging results led to an open-label, random, controlled, phase III study in 17 institutions in China [67]. The results showed 73% of patients (150/206) with AML (non-acute promyelocytic leukemia (APL)) in the HAA (HHT 2 mg m^-2^ d^-1^ for 7 days, Ara-c 100 mg m^-2^ d^-1^ for 7 days, and aclarubicin 20 mg d^-1^ for 7 days) group achieved CR, which was significantly higher than that in the DA (DNR 40–45 mg m^-2^ d^-1^ for 3 days and Ara-c 100 mg m^-2^ d^-1^ for 7 days) group (61%, 125/205). Patients in CR were offered two cycles of intermediate-dose Ara-c (2 g m^-2^ every 12 h for 3 days). A 35.4% of 3-year event-free survival was observed in the HAA group versus 23.1% in the DA group. These results suggested an HHT-based triple drug combination, especially the HAA regimen, is a treatment option for young, newly diagnosed patients with AML (Table 1).

HHT was also used in the treatment of patients with APL. In 1992, Xu et al. administrated all-trans-retinoic acid (ATRA) and low-dose HHT (1 mg d^-1^ for 10 days, interval 5-7 days to next cycle) to 25 patients with APL and the CR rate was 92% [68]. In the study of Liu et al., thirty-five patients with APL were treated with ATRA and low-dose HHT (0.5 ~ 1 mg d^-1^) was added when WBC > (5 ~ 10 × 10^9^/L) until WBC < 4 × 10^9^/L. The adverse effects related to ATRA were significantly reduced [69]. Studies of Lin et al. and Cao et al. concerning ATRA and AS2O3 treatment of patients with APL confirmed that the addition of HHT could shorten the time to CR and reduce the leukocyte stasis [70,71]. Yuan et al. evaluated the therapeutic effect of HHT plus ATRA by comparing with DNR plus ATRA as an induction regimen (HA or DA as post- remission consolidation regimen) in 115 cases (54 in the HHT group and 61 in the DNR group) of APL. The results showed that after induction therapy, 31.3% and l5.5% of patients in the HHT and DNR groups, respectively, were converted to PML-RAR α negative status detected with RT-PCR. No statistically significant difference was found on OS and EFS between the HHT group and the DNR group. This study demonstrated a comparable therapeutic effect of HHT and DNR on APL. HHT was also well tolerated and did not cause more serious adverse events than DNR [72] (Table 2). A recent study by Pei et al. showed that HHT in combination with ATRA and AS2O3 for newly diagnosed APL has a better efficacy, higher long-term survival and lower costs than idarubicine in combination with ATRA and AS2O3 [73]. Attractively, Liu et al. evaluated the cardiotoxicity of HHT and DNR in the treatment of APL when combined with ATRA in a single-centre, random, controlled study. The results showed HHT and DNR displayed similar cardiotoxicity, mainly ST-T changes and left-ventricular fractional decrease in some patients [74] (Table 3).

**Table 3:** Comparison of the cardiotoxicitv of homoharringtonine and daunorubicin in adults with acute promyelocytic leukemia

**Reference**	**Accumulative dose (mg.m**^**-2**^**)**	**Total(n)**	**Change of ST-T(n)**	**Nodal tachycardia (n)**	**Decrease of LVEF > 10% (n)**	**Increase of myocardial enzyme (n)**
**Liu 2012 [**[74]**]**	HHT(44)	27	3	4	8	0
	DNR(405)	28	5	5	7	0

*DNR* daunorubicin, *HTT* homoharringtonine, *LVEF* left ventricular ejection fraction.

HHT-based regimens were also effective to patients with relapsed and refractory AML. In a study performed by Fu et al., 27 AML patients who were NR or relapsed after DA treatment received HA treatment; 16 (59.25%) of them obtained CR. The results suggested HHT was active in the treatment of relapsed and refractory AML and there was no cross resistance between HHT and DNR [60]. Meng et al. treated 24 patients with refractory AML by a regimen combining HA with etoposide or teniposide, and 80% patients achieved CR [75]. Sensitization of leukemic cells with granulocyte colony-stimulating factor (G-CSF) can enhance the cytotoxicity of chemotherapy in AML. Therefore, many studies have been conducted to evaluate the effect of G-CSF priming combined with low-dose HA chemotherapy (HAG regimen) in patients with relapsed and refractory AML. In a study performed by Wei et al., the HAG regimen (G-CSF 200 μg m^-2^, beginning from one day prior to chemotherapy until WBC > 20 × 10^9^ L^-1^ for 14 ~ 28 d, Ara-C 10 mg m^-2^, 1/12 h, for 14 ~ 28 d, HHT l ~ 2 mg m^-2^ for 14 ~ 28 d) was used to treat 20 refractory AML patients, which resulted in a CR rate of 70% [76]. In another study, 36 AML patients (23 refractory and 13 relapsed) were treated with the similar HAG regimen (Ara-c 7.5 mg m^-2^ 12 hr^-1^ on days 1 ~ 14, HHT 1.5 mg m^-2^ on days 1 ~ 14, and G-CSF150 μg m^-2^ on days 0 ~ 14). Fifty percent of patients achieved CR with a median CR duration of 7.2 months [77]. In some similar studies reported, the CR rates of the HAG regimen varied from 43% to 52.2% in relapsed, refractory or hypocellular AML, the TRM of HAG regimen is low[78,79]. These studies suggested that the HAG regimen is highly effective for refractory or relapsed AML patients without severe side effects (Table 4).

**Table 4:** The summary of studies of HAG regimen for AML, high-risk MDS and MDS/AML

**Reference**	**Patients type**	**No.**	**CR, %**
**Wei 2006 [**[76]**]**	Refractory or relapsed AML	20	70.0
**Zhang 2008 [**[77]**]**	Refractory or relapsed AML	36	50.0
**Ji 2010 [**[78]**]**	Refractory or relapsed AML	37	46.0
**Gu 2011 [**[79]**]**	Refractory or relapsed AML	67	52.2
**Liu 2006 [**[80]**]**	Elderly AML	31	58.1
**Shu 2007 [**[84]**]**	MDS-RAEB	28	53.6
**Su 2008 [**[85]**]**	High-risk MDS or MDS/AML	33	46.7
**Wu 2009 [**[86]**]**	High-risk MDS or MDS/AML	32	46.9
**Wu 2011 [**[87]**]**	Elderly high-risk MDS or MDS/AML	33	57.6

*HAG* homoharringtonine + arabinoside + G-CSF, *CR* complete remission, *AML* acute myeloid leukemia, *MDS* myelodysplastic syndrome.

*MDS/AML* MDS evolving to AML.

The efficacy of priming HAG chemotherapy was also widely evaluated in elderly patients with AML. In a study performed by Liu et al., 31 elderly AML patients (aged 57–72) were treated with the HAG regimen (G-CSF 200 μg m^-2^, on days 1–14, HHT lmg m^-2^ on days 1–14, Ara-C 10 mg m^-2^, 1/12 h, on days 1–14), resulting in a CR rate of 58.1% and an OR rate of 80.6%, which were significantly higher than those (CR 32.4%; OR 55.9%) in the HA group (HHT 4 mg, on days 1–7, Ara-c 100 mg m^-2^, on days 1–7). The myelosuppression of the HAG regimen was milder than the HA regimen [80] (Table 4).

In the USA, a phase I trial conducted by Feldman et al. confirmed the HHT 4 mg m^-2^ for 7 days by continuous infusion in combination with Ara-c is safe and effective for patients with AML [81]. However, there was no further related report after this trial in the USA and clinical data of omacetaxine in the treatment of AML is still absent. To fully estimate the effect and toxicity of HHT and omacetaxine compared with DNR in the treatment of AML, especially to compare HA regimen with standard DA regimen, multiple-centre, randomized, controlled phase III trials are required.

### HHT clinical development in high-risk MDS or MDS evolving to AML (MDS/AML)

In China, harringtonine and HHT were also widely used to treat patients with high-risk MDS or MDS/AML. Cao et al. treated patients of MDS-RAEB or MDS/AML with low-dose harringtonine (0.5 ~ 1 mg, intravenously, daily or once every two days, for 10 ~ 15 days, with an interval of 5 ~ 10 days between the two cycles) during 1984–1989, CR was achieved in 4 of 13 patients [13]. Subsequently, Ji et al. reported a 50% (7/14) CR rate in patients with MDS-RAEB or MDS/AML treated with low-dose harringtonine (0.5 ~ 1 mg d^-1^, intravenously, for 10 ~ 15 days, with an interval of 7 ~ 10 days between the two cycles) [82]. In a phase II trial in the USA reported by Feldman et al., HHT was administered at a dose of 5 mg m^-2^ by 24-h continuous infusion daily for 9 days to patients with MDS or MDS/AML. CR was achieved in seven patients, and the OR rate was 28% (8/28). Significant myelosuppression was universal and resulted in a high incidence of induction deaths (13/28) caused by neutropenia-related infections [83].

The priming HAG regimen that was highly effective for refractory or relapsed AML was also widely used to treat high-risk MDS or MDS/AML (Table 4). In a study by Shu et al., 28 MDS-RAEB patients were treated with the HAG regimen, which resulted in a CR rate of 53.6% [84]. Similarly, Su et al. reported that 46.67% of 33 newly diagnosed patients with high-risk MDS or MDS/AML treated with one course of HAG as induction chemotherapy achieved CR, while the CR rate in the group of HA regimen was 33.3%. The difference was statistically significant between the two groups [85]. Meanwhile, Wu et al. reported a 46.9% CR rate in 32 patients with advanced MDS or MDS/AML after one course of HAG therapy [86]. Wu et al. also evaluated the efficacy and toxicity of the HAG regimen as induction chemotherapy for elderly patients with high-risk MDS or MDS/AML. The CR rate was 57.6% (19/33). The median OS was 15 months. Grade 3/4 thrombocytopenia occurred in 28% patients and neutropenia in 34%. No treatment-related deaths occurred during the induction therapy. The data suggest that the HAG priming regimen is effective and safe as an induction therapy for patients, including elderly patients, with high-risk MDS and MDS/AML [87]. These studies also suggested that stronger and alternative subsequent chemotherapy is necessary for patients achieved CR to maintain longer CR duration and better OS [84-87].

These data of HHT in the treatment high-risk MDS and MDS/AML were generally scattered and retrospective. So multiple-center prospective randomized trials are also needed to evaluate the effect and toxicity of HHT (or omacetaxine)-based regimens, especially HAG regimen in the treatment of high-risk MDS and MDS/AML.

## Summary

HHT, a plant alkaloid with antitumor properties originally identified nearly 40 years ago, has a unique mechanism of action compared with other antitumor drugs. HHT inhibits protein synthesis by competing with the amino acid side chains of incoming aminoacyl-tRNAs for binding to the A-site cleft in the peptidyl transferase center of the ribosome. HHT induces the rapid loss of a number of short-lived proteins regulating proliferation and cell survival of various cell lines from hematological malignancies, which triggers HHT-induced apoptosis. In addition, sHHT (omacetaxine) caused less damage to the environment and another potential advantage is its excellent bioavailability by the SC route, which provides patients with the opportunity to self-administer their therapy. Preclinical studies have proved the synergistic effect of HHT with other agents, such as IFN-a, Ara-C and imatinib. Data from preclinical studies also showed the remarkable effects of HHT and omacetaxine on LICs and imatinib-resistant Bcr-Abl mutants (including T315I). Clinical studies suggested that HHT is effective for patients with CML-CP, and HHT-based combination can improve clinical outcomes. Clinical studies also suggested that HHT and omacetaxine may provide an effective treatment for TKIs-resistant CML patients with Bcr-Abl mutations, including T315I, and could allow a safe TKIs rechallenge. Many studies in China showed that HHT-based combination therapy is highly effective in the treatment of young, newly diagnosed AML, with at least comparable results with that of DA regimen. Clinical studies also showed that HHT-based regimens, especially the HAG priming regimen, are well tolerated and effective in patients with relapsed and refractory AML, high-risk MDS, MDS/AML or elderly patients with AML. The main adverse effects of HHT in the treatment of AML and MDS were myelosuppression and cardiotoxicity which were at least not more severe than DNR. But HHT was rarely used for the treatment of AML and MDS outside of China and there were also no clinical data of omacetaxine in the treatment of AML and MDS up to date.

Further studies should assess the suitability of combining HHT with TKIs and/or other agents in an attempt to improve current salvage regimens for patients with CML. In order to avoid Bcr-Abl mutating and cure the disease by killing LICs, clinical trials of the combination of HHT or HHT analogs with TKIs in the treatment of patients with newly diagnosed CML are also deserve to carry out. Studies should be performed to expand and validate the experiences of HHT in the treatment of AML and MDS in China with multiple-center prospective randomized trials.

The generation of HHT analogs with improved toxicity profiles and perhaps with oral bioavailabity should also be explored [88].

## Competing interests

The authors declare that they have no competing interests.

## Authors’ contributions

Both authors read and approved the final manuscript.
